# Fluorescent Carbon Dots-Antibiotic Nanocomposite Drug for the Eradication of Multidrug-Resistant Bacteria

**DOI:** 10.1021/acsomega.5c08753

**Published:** 2026-02-01

**Authors:** Vijay Bhooshan Kumar, Yakov Shalom, Ze’ev Porat, Ehud Banin, Aharon Gedanken

**Affiliations:** † Bar Ilan Institute for Nanotechnology and Advanced Materials, Department of Chemistry, Bar-Ilan University, Ramat-Gan 52900, Israel; ‡ Advanced Engineering, Product Development, Ashok Leyland Limited, Technical Centre, Vellivoyalchavadi, Manali New Town, Chennai, Tamil Nadu 600103, India; § Bar Ilan Institute for Nanotechnology and Advanced Materials, Mina and Everard Goodman Faculty of Life Sciences, Bar-Ilan University, Ramat Gan 52900, Israel; ∥ Department of Civil and Engineering, Ben-Gurion University of the Negev, Be’er Sheva 84105, Israel

## Abstract

Hospital-acquired infections are a growing concern for healthcare providers worldwide. Recently, attention has been devoted to new and emerging nanoparticle-based materials that can help antibiotics destroy multidrug-resistant bacteria. In this work, we conjugated two commercial antibiotics, ampicillin or chloramphenicol, with carbon dots (CDs), forming novel nanoantibiotic-CDs nanocomposites in order to enhance their activity against multidrug-resistant bacteria. These CDs-antibiotics composites were about 4–7 nm in size and were prepared by sonication of poly­(ethylene glycol) (PEG)-400 and the antibiotic drug without using a catalyst. The antibacterial activity of the composites was tested and showed a 4-fold effect in the case of chloramphenicol and at least a 2-fold effect in the case of ampicillin, as compared to the unbound drugs. This method may provide a simple strategy to bring back the use of antibiotics that are no longer clinically relevant due to the increase in antibiotic resistance.

## Graphical abstract

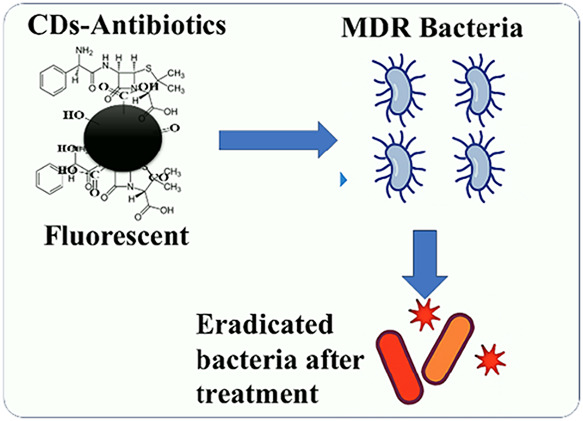

## 1 Introduction

Global healthcare growth in the past 100 years has been unprecedented, and the primary reason can be attributed to innovations in allopathy, particularly antibiotics. Recently, there has been a growing appeal for the development of strong resistant bacteria against most commercially available antibiotics.^1^ However, a challenging problem that arises is that the number of new antibiotics expected to enter the market is limited.^2^ The use of heavy doses of antibiotics, combined with person-to-person spread of bacteria, has significantly increased antibiotic resistance due to genetic mutation,^1,3^ a problem that is increasing in severity.^4^ Nowadays, one of the major investigation in this field is focused on developing new antimicrobial drugs or agents, with one objective of killing such resistant bacteria using nanomaterials.^5–7^ Recently, carbon-based nanomaterials have emerged as a new area of research that shows enormous potential for a significant impact on biomedical science. Mostly, the carbon derivatives nanomaterials like carbon quantum dots (CQDs), carbon dots (CDs), carbon nanotubes, and graphene, were shown to augment efficient killing of normal and antibiotic-resistant bacteria.^8–11^ Carbon dots are a class of zero-dimensional materials that exhibit discrete quantized energy levels and different densities of states.^12^ The CDs have outstanding fluorescence, excellent aqueous solubility, biocompatibility, biodegradation ability, imperceptible cytotoxicity, easy functionalization, and environmental friendliness.^13–16^ Much effort has been devoted to the development of new synthetic procedures for the large-scale and inexpensive production of nanoantibiotics. Studies have demonstrated that nanomaterial-based antibiotics can be obtained from a variety of carbonaceous materials.

CDs were synthesized by methods such as chemical vapor deposition,^17^ sonochemical synthesis^16,18^ microwaves^19^ and hydrothermal method.^20,21^ Remarkably, the characteristic properties of CDs that are prepared by the hydrothermal method are partially dependent on the starting raw materials, such as amino acids, polymers, sugars, proteins, or carbohydrates. However, the previously reported quantum yield of the CDs and their photostability are still lower than 16%, which is relatively low for long-term biological processes.^18^ Chen et al. reported the synthesis of CDs combined with ZnO via a hydrothermal one-step method, and observed that CDs/ZnO nanocomposite demonstrated significantly improved antimicrobial activity compared to CDs alone, primarily due to enhanced reactive oxygen species generation upon illumination of light.^22^ Zare et al. published a review on emerging mechanisms for enhancing antibacterial effectiveness, such as antimicrobial phototherapy, enzymatic cascade activity, phytochemical therapy, and synergistic effects in combination with antimicrobial agents and herbal extracts.^23^ These include bacterial detection and dressings for bacteria-infected wounds, ocular, periodontal, bone and implant-related infections.^23^ Arsalani et al.^24^ prepared poly­(ethylene glycol) passivated CDs (CDs-PEG) from gelatin and PEG by microwave pyrolysis. These were found to be good anticancer drug nanocarriers for the drug methotrexate (MTX), more efficient than free MTX in the inhibition of tumor growth. Chung and Zhang^25^ also used the microwave method to synthesize fluorescent CDs composed of glucosamine bonded to the copolymer chitosan–PEG. This product was found to be nontoxic and allowed quantitative fluorescence imaging that was used to analyze the population of treated cells. Conjugation with the drug doxorubicin (DOX) yielded an efficient cancer cell killer that was able to deliver the chemotherapeutic agent.

CDs can be used as a potent antibacterial agent themselves or as a nanocarrier to deliver antibiotic molecules more effectively. Some recent studies reported that CDs can kill microbes by disrupting their cell walls and membranes, generating reactive oxygen species (ROS), which damage cellular components, or acting as nanoenzymes.^26–28^ When combined with antibiotics, they can serve as nanocarriers that improve delivery directly into target cells, can enhance the stability and efficacy of the antibiotics, and can reduce the drug resistance, thus creating broad-spectrum activity and a lower risk of developing drug resistance compared to the antibiotic alone.^29,30^ Several studies reported on the activity of CDs and CQDs against *Pseudomonas aeruginosa*,^31,32^ *Bacillus subtilis* cells^33^ and *Escherichia coli* bacteria.^22^ Gayen demonstrated that CDs are a kind of mystic star in the world of nanoscience^34^ by using them for targeted therapy against bacterial infections.^11^ Yang et al. showed that CDs-doxorubicin conjugated nanoparticles show a green fluorescent for bioimaging and enhanced intracellular biomedical applications.^35^ Liu et al. highlighted the antimicrobial activity of ZnO-CDs nanocomposites for enhancing the antibacterial activity against *E. coli*.^31^ Otis et al. demonstrated that CDs are very selective to *P. aeruginosa* bacteria.^32^ They also showed that the surface of biocompatible CDs plays a major role in targeting the CDs to *P. aeruginosa* cells, affecting cell staining, bacterial growth inhibition, and bacterial biofilm formation disruption.^32^ Dong et al. investigated the antimicrobial effects of CDs in combination with antimicrobial reagents, including H_2_O_2_, Na_2_CO_3_, and acetic acid.^8^ They observed that the minimal inhibitory concentration (MIC) of CDs, which is the lowest concentration that prevents visible in vitro growth of bacteria, was 64 μg/mL on both Gram-negative bacteria *E. coli* cells and Gram-positive bacteria *B. subtilis* cells.^8^ The combination of CDs with Na_2_CO_3_ or AcOH did not show synergistic effects, but the combination of CDs with H_2_O_2_ did show a synergistic antimicrobial effect.^8^ However, H_2_O_2_ cannot be used in treating humans due to its toxicity. There are also few recent reports detailing the antibacterial activity of CDs^36,37^ and a CDs-Ag nanocomposite.^38^ Although several studies have reported on CDs-antibiotic systems^30,35,39–41^ and their antibacterial properties, comprehensive investigations into their synergistic activity against multidrug-resistant bacterial strains remain limited.

In recent years, we prepared a series of CD nanomaterials using sonochemical and hydrothermal methods. We also synthesized metal/nonmetal-doped CDs and CDs-conjugated nanomaterials and examined their employment in a broad range of applications, such as ointments,^42^ antibacterial drugs,^43^ lubricants,^44^ cell labeling,^45,46^ catalysis,^46^ polymerization,^47^ drug delivery agents,^24,25^ and for neural tissue engineering. Recently, we synthesized Ga-doped C-dots (Ga@CDs), which were found to be efficient for killing the opportunistic pathogen *P. aeruginosa* bacteria. In this study, we synthesized and characterized the antimicrobial activity of CDs-antibiotics nanoparticles. We utilized a two-step sonochemical approach, continuing the one-step synthesis process of CDs.^16^ Here we describe the conjugation of two different antibiotics: ampicillin (Amp.) and chloramphenicol (Chlor.) with sonochemically synthesized CDs, and show increased antimicrobial activity against multidrug-resistant bacteria. The chemical and physical properties of the CDs-antibiotic nanoparticles were also studied by various techniques, including transmission electron microscopy (TEM), fluorescence spectroscopy, and UV–visible analysis. The antidrug-resistant bacteria of CDs-antibiotic nanoparticles, as a function of concentration, was compared with that of CDs and antibiotics alone. Our results highlight the potential use of CDs-antibiotic nanoparticles as antimicrobial agents for multidrug-resistant bacteria.

## 2 Experimental Section

### 2.1 Chemicals

Poly­(ethylene glycol)-400 (PEG-400), ammonium hydroxide, ampicillin, chloramphenicol, and quinine sulfate were purchased from Sigma-Aldrich (St. Louis, MO, USA). Ethanol, acetone, and isopropanol were obtained from Romical Ltd. (Jerusalem, Israel). 5,5-dimethyl-1-pyrroline *N*-oxide (DMPO) was used as “spin trap” to detect free radicals by ESR. Deionized water (DI water) (18.3 MU) was used throughout the experiments.

### 2.2 CDs Preparation and Characterization

The preparation of water-soluble CDs was done via a modified sonochemical method using PEG-400, as described previously.^16^ In brief, 20 mL of PEG-400 (99.98% purity) were added to a 50 mL beaker, which was dipped in Si-oil bath at 70 °C and sonicated for 3 h. The appearance of a light-yellow color indicated the formation of a suspension of CDs. This was transferred into a dialysis bag for 24 h in deionized water to remove all of the unreacted PEG-400.

### 2.3 Synthesis of CDs-Antibiotics Nanoparticles

The sonochemically synthesized CDs (200 mg/mL) and the aqueous solution of chloramphenicol (100 mg/mL) were mixed and sonicated for 15 min. The resulting product was subjected to dialysis using a membrane with a molecular weight cutoff (MWCO) of 3 kDa for 48 h against deionized water, with frequent water changes every 6 h. This step effectively removed unreacted antibiotic molecules and small byproducts. The dialyzed solution was then centrifuged at 12,000 rpm for 15 min to eliminate any aggregates or insoluble residues. The same procedure was used to prepare the CDs-ampicillin nanoparticles, as depicted schematically in Figure 1. The supernatants containing purified CD-antibiotic hybrids were lyophilized and stored for further analysis.

**Figure 1:**
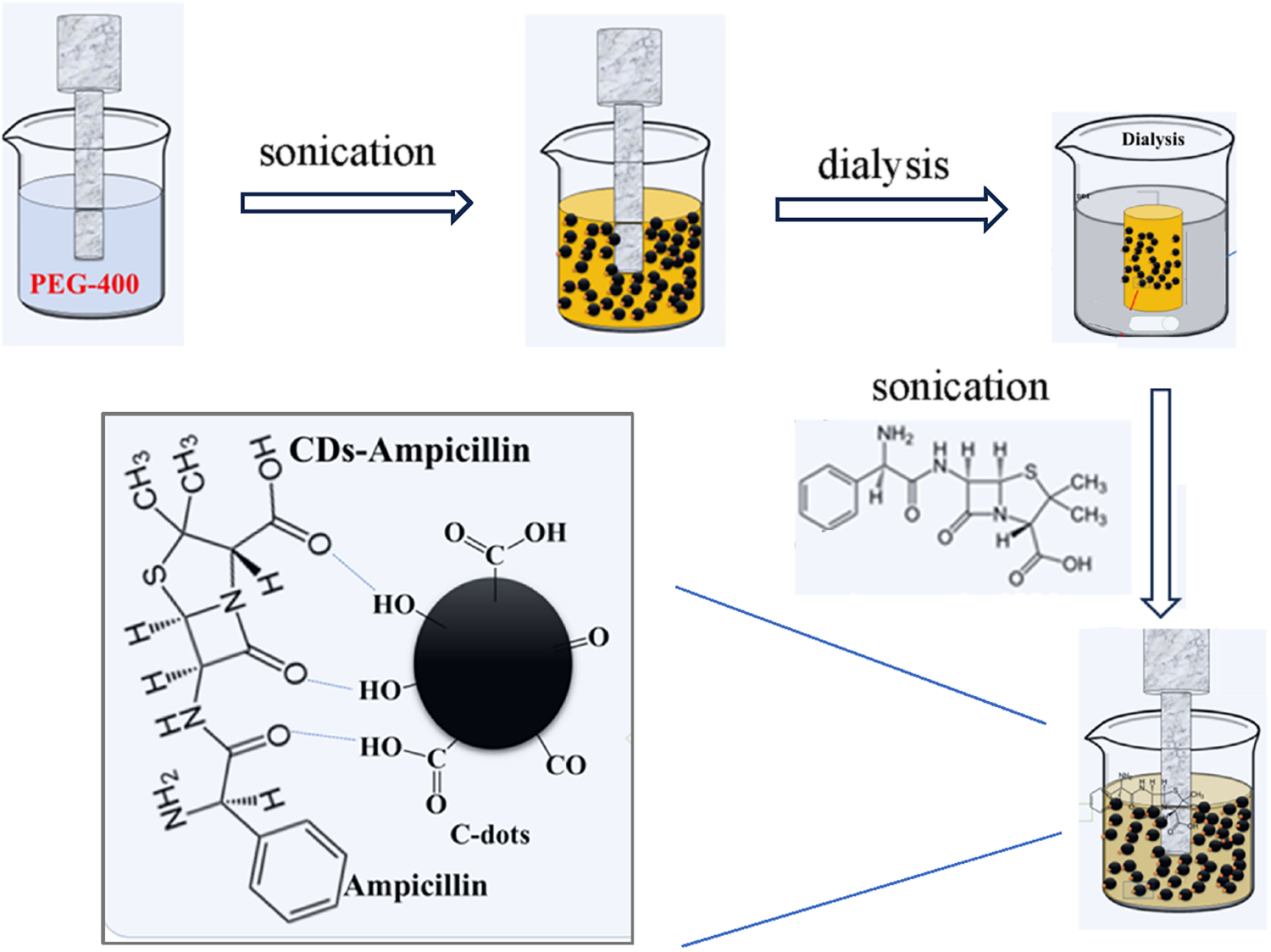
Schematic presentation of the synthesis process of CDs-antibiotic nanoparticles and possible binding sites.

### 2.4 Analysis of the CDs-Antibiotics Nanoparticles

The fluorescence of the CDs-antibiotic nanoparticles was measured by a fluorescence spectrophotometer (Varian Cary Eclipse, Varian GmbH, Darmstadt, Germany). UV–vis absorption spectra of an aqueous solution of CDs-antibiotics nanoparticles were measured using a Cary 100 spectrophotometer (Varian), operated by Lab Sphere software. The morphology and crystalline properties of CDs-antibiotics nanoparticles were analyzed by high-resolution transmission electron microscopy (HRTEM) using a JEOL 2100 (JEOL USA, Inc., Peabody, MA, USA) microscope that was operated at 200 kV. The samples for the HRTEM analysis were prepared by adding a few drops of CDs-antibiotics nanoparticles to 1 mL of isopropanol and mixing in an ultrasonic bath for 2 min. A droplet of the resultant suspension was applied on a silicon-coated copper grid and then dried overnight under vacuum at 25 °C. Particle size distribution was measured by dynamic light scattering (DLS), using a ZetaSizer Nano-ZS instrument from Malvern Instruments Ltd., based in Worcestershire, U.K. Raman spectroscopy was done with a Horiba spectrometer LabRAM micro-Raman, France, at an excitation wavelength of 532 nm. Fourier transform infrared (FTIR) spectroscopy was done with a Bruker ATR Advance system (Bruker AXS GmbH, Karlsruhe, Germany).

The ROS half-life of CDs, CDs-antibiotics nanocomposites, is too short compared to the electron paramagnetic resonance (EPR) time scale; therefore, it must be trapped and transformed into a more stable radical species. The ROS generation was analyzed using DMPO as a spin trap to form the spin adduct DMPO–OH. An aqueous suspension of the designed formulations at 100 mM was added to a 9 : 1 mixture of DDW and DMPO. The solutions of the samples were sucked into a capillary quartz tube (0.8 mm i.d.) and sealed at both ends by a plastic Critoseal (Thermo Fisher Scientific). The sealed capillary was then placed into a quartz tube, which was inserted into the rectangular EPR cavity (ER code 4122SHQ). The EPR measurements were performed at the following conditions: microwave power of 20 mW; scan width of 100 G; resolution of 1024; gain of 60; sweep time of 60 s; number of scans of 2; modulation frequency of 100 kHz; and modulation amplitude of 1 G. The radical intensity was compared by applying the double integration of the spin adduct signal using the EPR 2.6b.58 acquisition version.

### 2.5 Bacterial Culture Preparation

Cultures of Dh5a–ampicillin R and BL21 chloramphenicol R were grown in Luria–Bertani (LB) broth media at 37 °C for 15–17 h. The overnight bacterial culture growth was quantified by OD_595nm_ and diluted to 10^8^ CFU/mL. The cells were then washed twice with DDW. Samples were diluted (with DDW) to achieve the same concentrations of the Zn and Cu ions. A final volume of 1.5 mL, consisting of the 10^7^ CFU/mL bacterial suspensions and the tested samples, was incubated for up to 50 min at 37 °C with 220 rpm agitation. A 100-μL sample, taken at 0, 10, 25, 35, and 50 min, was subject to a 10-fold serial dilution in saline and plated on LB agar plates for viable cell counting. The plates were incubated overnight at 37 °C, and the CFU/mL was determined.

### 2.6 Cell Culture and Cytotoxicity Assay

HeLa cells were cultured in Dulbecco’s modified Eagle’s medium (DMEM, Gibco) supplemented with 10% fatal calf serum (FCS). Where indicated, casamino acids–DMEM were used instead of DMEM at the relevant concentration. HeLa cells were seeded at 10^4^ cells per well in a 96-well plate (Greiner Bio-One) and incubated at 37 °C in a humidified atmosphere of 5% CO_2_. After 24 h, the HeLa cells were washed twice with cDMEM and then supplemented in with one of the following solutions at the relevant concentration: CDs; antibiotics; CDs-antibiotic conjugation and their pristine antibiotics, DDW, cDMEM (negative control), and lysis solution (positive control). This is the type of MTT assay (MTT assay stands for 3-(4,5-dimethylthiazol-2-yl)-2,5-diphenyltetrazolium bromide assay) and was performed using human cervical carcinoma (HeLa) cells. Cells were seeded in 96-well microtiter plates and allowed to attach overnight at 37 °C under 5.0% CO_2_. The cells were then washed with pH 7.4 phosphate-buffered saline (PBS) buffer and incubated with an MTT solution for 3.5 h. Thereafter, the MTT reagent was discarded, and 100 μL of dimethyl sulfoxide (DMSO) was added to each well to dissolve formazan crystals. The absorbance was recorded by using a microplate reader at 570 nm. Control experiments were conducted in the absence of CDs; antibiotics; CDs-antibiotic conjugation, and their pristine antibiotics. All of the experiments were carried out in quadruplicates.

## 3 Results and Discussion

### 3.1 Characterization of the CDs-Antibiotics Nanoparticles

The water-soluble CDs-antibiotics nanoparticles were synthesized by sonicating a mixture of purified CDs with an aqueous solution of the antibiotics (chloramphenicol or ampicillin). A high-power ultrasonic was employed to facilitate the reaction. The sonication process was conducted continuously (60–100% duty cycle) for a duration of 3 h using a 13 mm titanium alloy probe to ensure efficient energy transfer and uniform dispersion. Throughout the synthesis, the reaction temperature was maintained at 70 °C using a thermostatic water bath to promote consistent nucleation and growth of the nanoparticles. This controlled sonochemical approach enabled the formation of spherical CDs. The CDs contain various functional groups on their surface, such as −COOH, −OH, −O–, −COOR, and −CO, which could serve as binding sites to both kinds of antibiotic molecules and thus form the hybrid CDs-antibiotics nanoparticles. The second step, i.e., the conjugation of antibiotics onto the CDs, was also done by ultrasonic irradiation but at 0 °C, to avoid structural changes of the antibiotic molecules.

The conjugation of carbon dots with the antibiotics ampicillin and chloramphenicol was confirmed by both UV–visible (UV/vis) absorption spectroscopy and Fourier transform infrared (FTIR) spectroscopy. The UV/vis absorption spectra of the CDs-Amp and CDs-Chol nanoparticles (Figure 2a,b) appear as superpositions of the separate spectra for the CDs and each of the antibiotic molecules. The CDs-Amp conjugates exhibited two distinct absorption peaks at approximately 292 and 351 nm,^16^ which may arise from the two components: The bare CDs displayed a single absorption peak at 282 nm and a shoulder at ca. 325 nm with a broad tail, while ampicillin showed two peaks at 287 and 340 nm. The observed red shift in the absorption peaks after conjugation suggests electronic interactions and surface modification of CDs with ampicillin molecules. The spectrum for CDs-Chlor nanoparticles (Figure 2b,d) exhibits a single broad absorption peak at ca. 280 nm, as for Chlor. alone, and a slight red shift for CDs alone, which may indicate surface modification.

**Figure 2:**
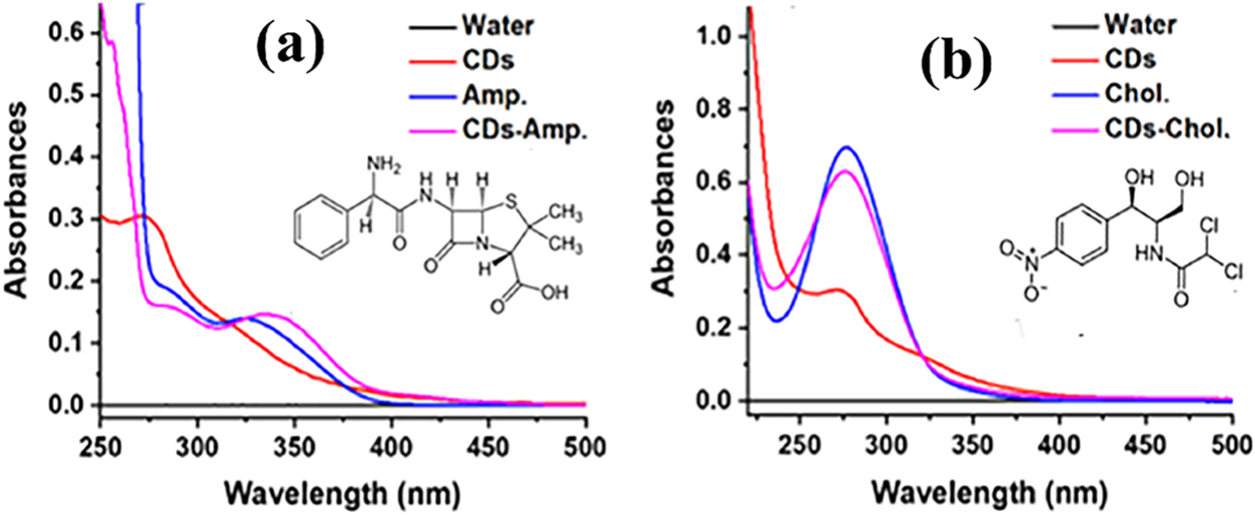
UV/vis spectroscopy of the (a) CDs-ampicillin as compared with pristine CDs, water, and ampicillin. (b) CDs-chloramphenicol as compared with pristine CDs, water, and chloramphenicol.

Figure 3 presents the fluorescence spectra for aqueous solutions of CDs, ampicillin, chloramphenicol, CDs-ampicillin, and CDs-chloramphenicol at different excitation wavelengths (300–500 nm), showing emission signals in the range of 400–610 nm. The aqueous solution of ampicillin showed a clear fluorescence signal (Figure 3b), whereas that of chloramphenicol showed almost no fluorescence (Figure 3c). CDs-Amp (Figure 3d) and CDs-Chlor (Figure 3e) nanocomposites yielded stronger signals than those of the bare CDs (Figure 3a). Moreover, the quantum yields of both CDs-antibiotics nanoparticles were around 37% (360 nm excitation wavelength), which is much more intense than that of bare CDs (16%).^16,38^ The enhancement of the fluorescence signals for the two nanocomposites, compared to that of pristine CDs, indicates the formation of new kinds of species. Moreover, the bimodal shape of the signals is also an indication of the conjugation of the antibiotic molecules with CDs, having two types of adsorption bonding sites. Visual observation of the fluorescence shows a clear enhancement of the CDs-Chlor. and CDs-Amp. in comparison to the pristine molecules (Figure 4). This also indicates their conjugation to carbon dots.

**Figure 3:**
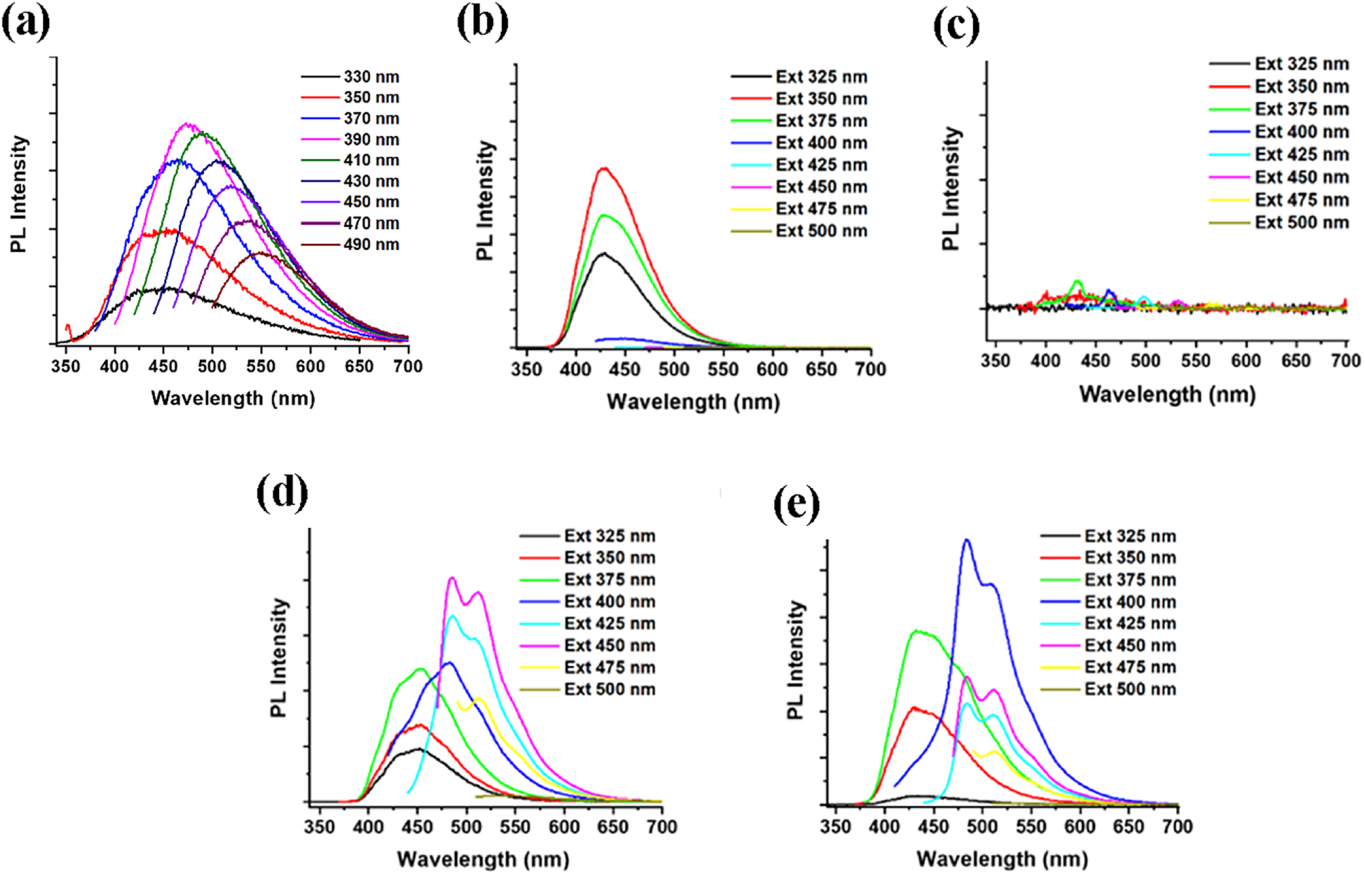
Fluorescence spectra, recorded in aqueous solutions of (a) pristine CDs, (b) ampicillin, (c) chloramphenicol, (d) CDs-ampicillin, and (e) CDs-chloramphenicol. Each sample was recorded at various excitation wavelengths, as indicated. Photoluminescence (PL) intensity scale is similar for all curves.

**Figure 4:**
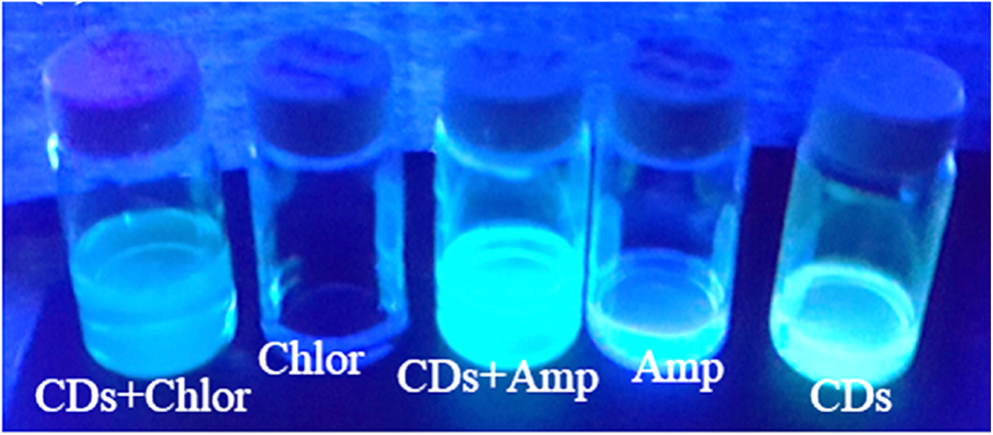
Visual presentation of the fluorescence of various samples under UV light.

The morphology and structural characteristics of the CDs and CDs-antibiotic hybrid nanoparticles were investigated by using transmission electron microscopy (TEM). It revealed that all three species had spherical shapes, in the size distribution range of 3–9 nm (Figure 5b,e,h), but the two hybrid nanoparticles had slightly larger average diameters. This modest increase in particle size is consistent with the surface functionalization and is in good agreement with our previous reports.^48^ Selected-area electron diffraction (SAED) demonstrated some crystallinity of the hybrid nanoparticles (Figure 5c,f,i), indicated by the presence of lattice fringes, whereas no crystallinity was detected in the pristine CDs, as the SAED exhibited just diffuse diffraction rings. The interplanar *d*-spacings (of the CDs-Amp and CDs-Chol nanoparticles) were calculated from the selected-area electron diffraction patterns and found to be approximately 0.21 nm. This is typical for graphitic carbon-based nanostructures and suggests the retention of a partially ordered carbon framework after conjugation. Energy-dispersive X-ray spectroscopy (EDS) analysis of the elemental composition of both CDs-Amp and CDs-Chlor nanoparticles revealed only the presence of carbon, oxygen, and nitrogen.

**Figure 5:**
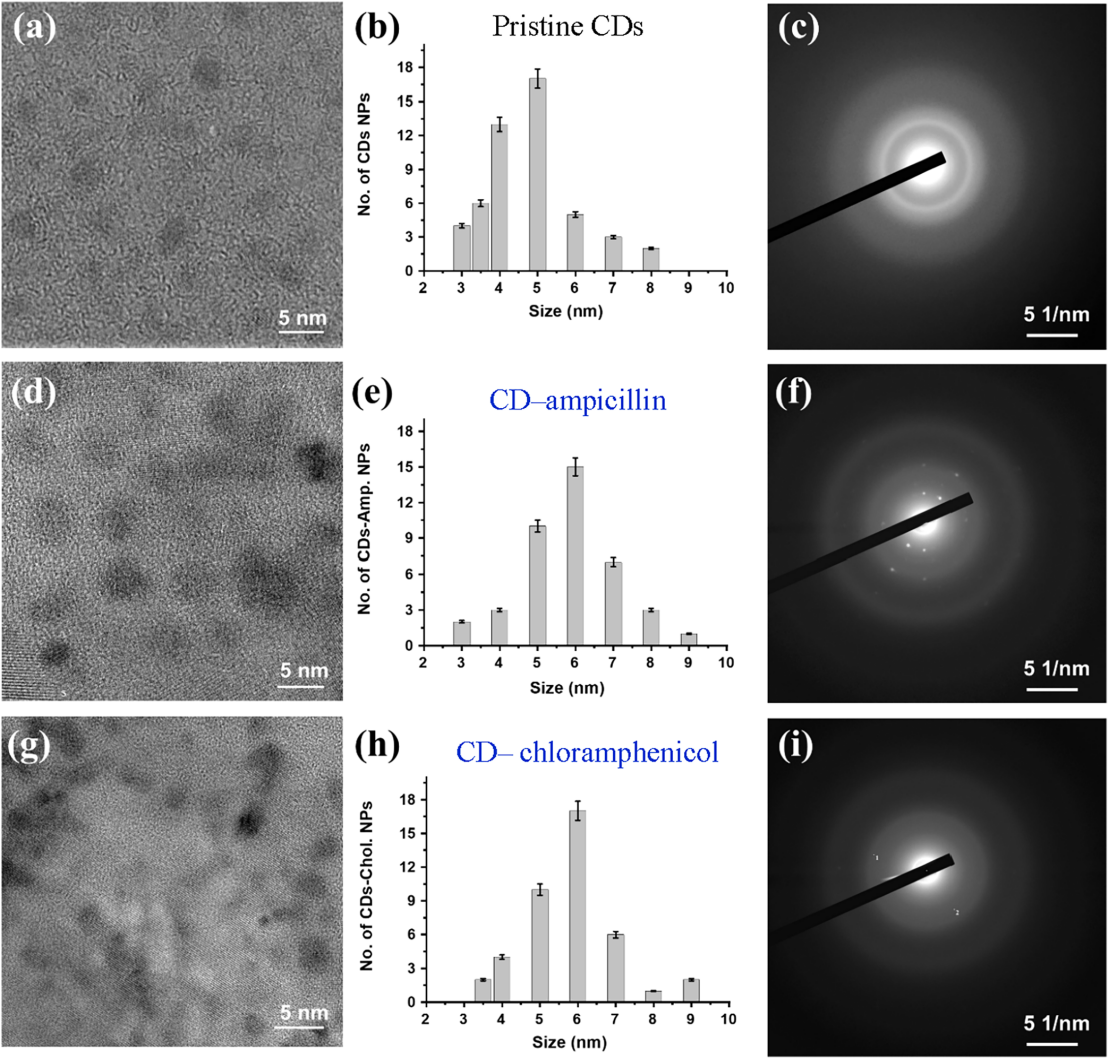
Morphological characterization of CD-based materials using HRTEM: (a) TEM image, (b) particle size distribution, and (c) SAED pattern of pristine CDs; (d) TEM image, (e) particle size distribution, and (f) SAED pattern of CD-Amp. composite nanoparticles; and (g) TEM image, (h) particle size distribution, and (i) SAED pattern of CD-Chlor. composite nanoparticles.

FTIR analysis can provide direct evidence of chemical interactions between the surface functional groups of CDs and the functional groups of the antibiotics. In general, the FTIR spectra for CDs-Amp. and CDs-Chlo. appear as superpositions of their single components. Specifically, the spectrum of the pristine CDs displayed characteristic absorption bands corresponding to surface groups −COOH (∼1700 cm^–1^), −OH (3200–3600 cm^–1^), and −NH_2_ (∼2900 cm^–1^) (Figure 6a).

**Figure 6:**
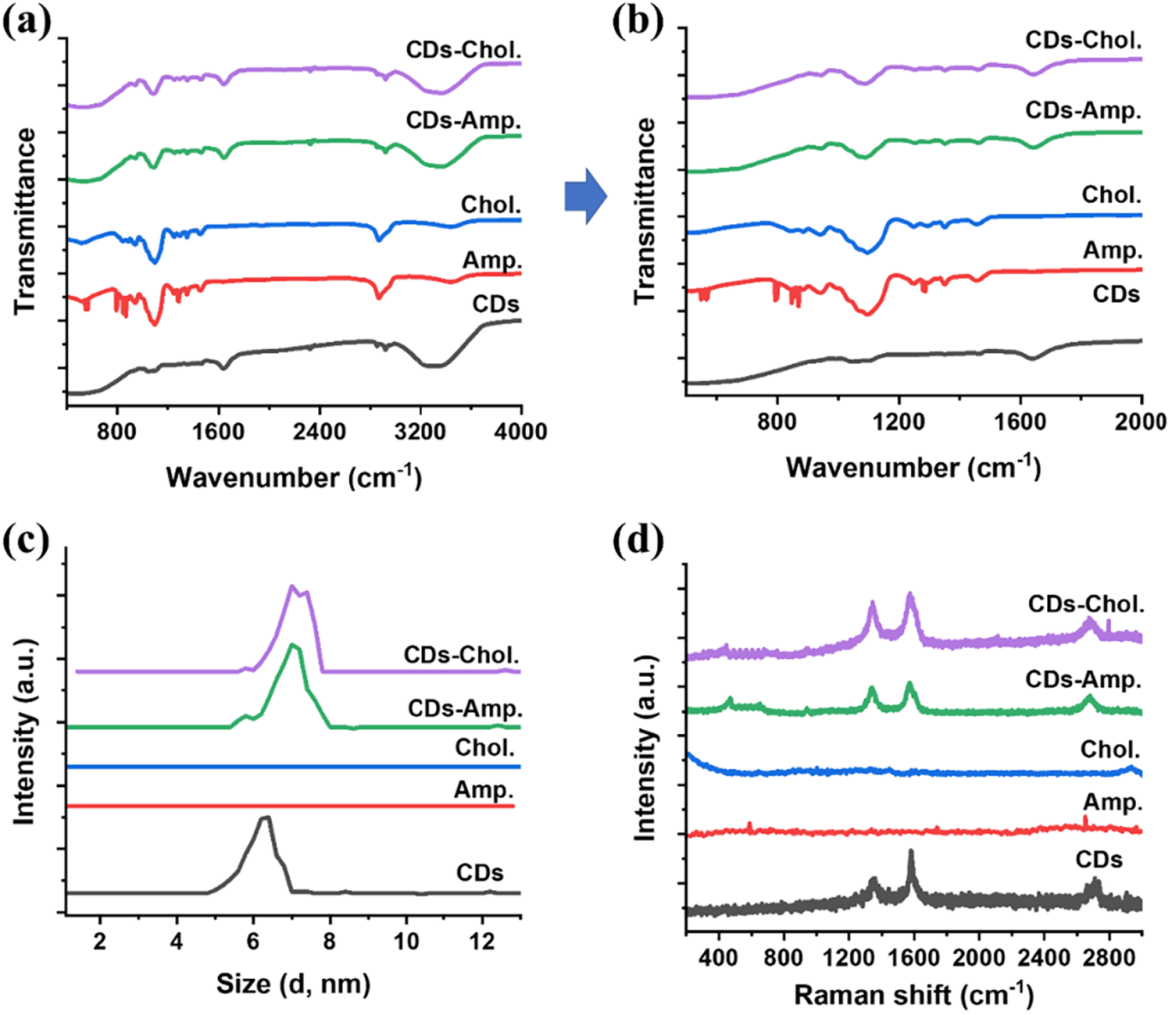
Comparative analyses of the CDs, the antibiotic molecules, and the conjugated products. (a, b) FTIR spectra. (c) DLS measurement for particle size distribution. (d) Raman spectra for carbon structure and banding properties of nanocomposites.

After conjugation, several changes were observed in the spectra of the CDs-antibiotic hybrids with respect to those of the pristine antibiotic molecules. For example, the broad O–H stretching vibration band around ∼3400 cm^–1^ showed a slight blue shift, indicating altered hydrogen bonding environments. Nevertheless, the comparative analysis of FTIR spectra also reveals the differences in the amide I and II regions as well as in the aromatic C=C stretching bands, which are consistent with the respective molecular signatures of ampicillin and chloramphenicol (Figure 6a,b). More notably, the C=O stretching vibration of carboxylic acid groups at ∼1700 cm^–1^ in bare CDs appeared now in the spectra for conjugated CDs-Amp. and CDs-Chol., also with a slight blue shift. These spectral changes, in combination with red shifts in UV/vis spectra (Figure 2) and altered fluorescence emission profiles (including both intensity changes and peak shifts), strongly support the conclusion that both covalent (e.g., amide bond formation) and noncovalent (e.g., hydrogen bonding, π–π stacking) interactions occurred during the conjugation process. The more prominent role of the −COOH groups can be attributed to their higher chemical reactivity under coupling conditions, while −OH groups likely contributed to secondary interactions such as hydrogen bonding. Collectively, the spectroscopic data confirm the successful grafting of antibiotic molecules onto the CD surface and the formation of stable CDs-antibiotic hybrid nanoparticles with modified electronic and structural properties.

Particle size distribution measurements of the carbon dots and their antibiotic-functionalized nanocomposites were performed by a dynamic light scattering (DLS) analysis (Figure 6c). It revealed that all three samples exhibited particle sizes ranging from 3 to 7 nm, but the CDs-Amp. and CDs-Chol. nanocomposites displayed slightly larger average diameters compared to pristine CDs. This modest increase in size is indicative of surface functionalization and aligns well with the particle size histograms obtained from HRTEM (Figure 5b,e,h). Subsequent to functional group characterization via FTIR (Figure 6a) and particle size analysis by DLS (Figure 6c), we performed Raman spectroscopy on CDs, CDs-Amp., CDs-Chol., and the free antibiotics ampicillin and chloramphenicol (Figure 6d). The Raman spectra of CDs and their antibiotic-functionalized counterparts exhibited a prominent graphitic G-band at 1565 cm^–1^ and a comparatively weaker D-band at 1372 cm^–1^, indicative of significant graphitization across all samples. Interestingly, the Raman signals of CDs-Amp. and CDs-Chol. were slightly more intense than those of pristine CDs, suggesting enhanced local electromagnetic field effects.

#### 3.1.1 Mechanism of CDs and CDs-Antibiotic Synthesis

The formation of CDs from poly­(ethylene glycol) (PEG-400) via sonochemical synthesis is driven by a series of rapid, high-energy transformations. When PEG-400 is exposed to ultrasonic irradiation, the liquid medium undergoes acoustic cavitation, a phenomenon in which microscopic bubbles form, grow, and collapse violently. This collapse generates extreme localized conditions, with temperatures reaching approximately 5500 K and pressures around 500 atm, albeit for microseconds.^49,50^ These conditions can trigger intermolecular dehydration and carbonization of PEG molecules. Although PEG has a low vapor pressure, meaning that only a small fraction vaporizes into the collapsing bubble, the surrounding liquid shell, roughly 200 nm thick, which experiences intense heat to make CDs nanomaterials.^49^ This thermal energy facilitates cross-linking reactions among PEG chains, leading to the breakdown of the polymeric chains and the formation of carbon-rich clusters. These clusters nucleate and grow into fluorescent carbon nanoparticles in the form of CDs, stabilized by residual PEG fragments or oxygen-containing functional groups on their surface. The result is a colloidal suspension of CDs with high stability and luminescence. To functionalize these CDs with antibiotics, a straightforward postsynthesis method was employed as described in the Section 2. During the second sonication step, noncovalent interactions such as hydrogen bonding, π–π stacking, and electrostatic forces facilitate the adsorption of antibiotic molecules onto the surface of the CDs. These interactions were enhanced by the presence of functional groups such as hydroxyl, carboxyl, and carbonyl on the CDs, which were introduced during the carbonization process.

### 3.2 Cell Viability and Antibiotic Activity

#### 3.2.1 HeLa Cell Viability Study

The MTT assay was performed in vitro to measure the cytotoxicity of CDs, CDs-ampicillin, and CDs-chloramphenicol nanocomposites. The viability diagrams for CDs nanomaterials show low toxicity even at 500 μg/mL (Figure 7a). When exposed to 200 μg/mL of CDs-ampicillin and CDs-chloramphenicol nanocomposites, the viability of the HeLa cells was ∼93% (Figure 7b) and 90% (Figure 7c), respectively. Although, as expected, the conjugation of CDs with antibiotic molecules resulted in slightly lower cell viability, as compared to pristine CDs, CDs-ampicillin and CDs-chloramphenicol nanocomposites show low cytotoxicity with respect to pristine ampicillin (Figure 7d) and chloramphenicol (Figure 7e). Additionally, it was observed that the pristine forms of the antibiotics exhibited relatively high levels of toxicity when compared to their corresponding CDs-antibiotic nanocomposites. This finding highlights the advantage of conjugating antibiotics with carbon dots, as the resulting nanocomposites, specifically CDs-ampicillin and CDs-chloramphenicol, demonstrated significantly reduced cytotoxicity. The lower toxicity profile of these nanocomposites suggests that they may provide a safer alternative for biomedical applications, offering therapeutic efficacy while minimizing harmful effects on healthy cells.

**Figure 7:**
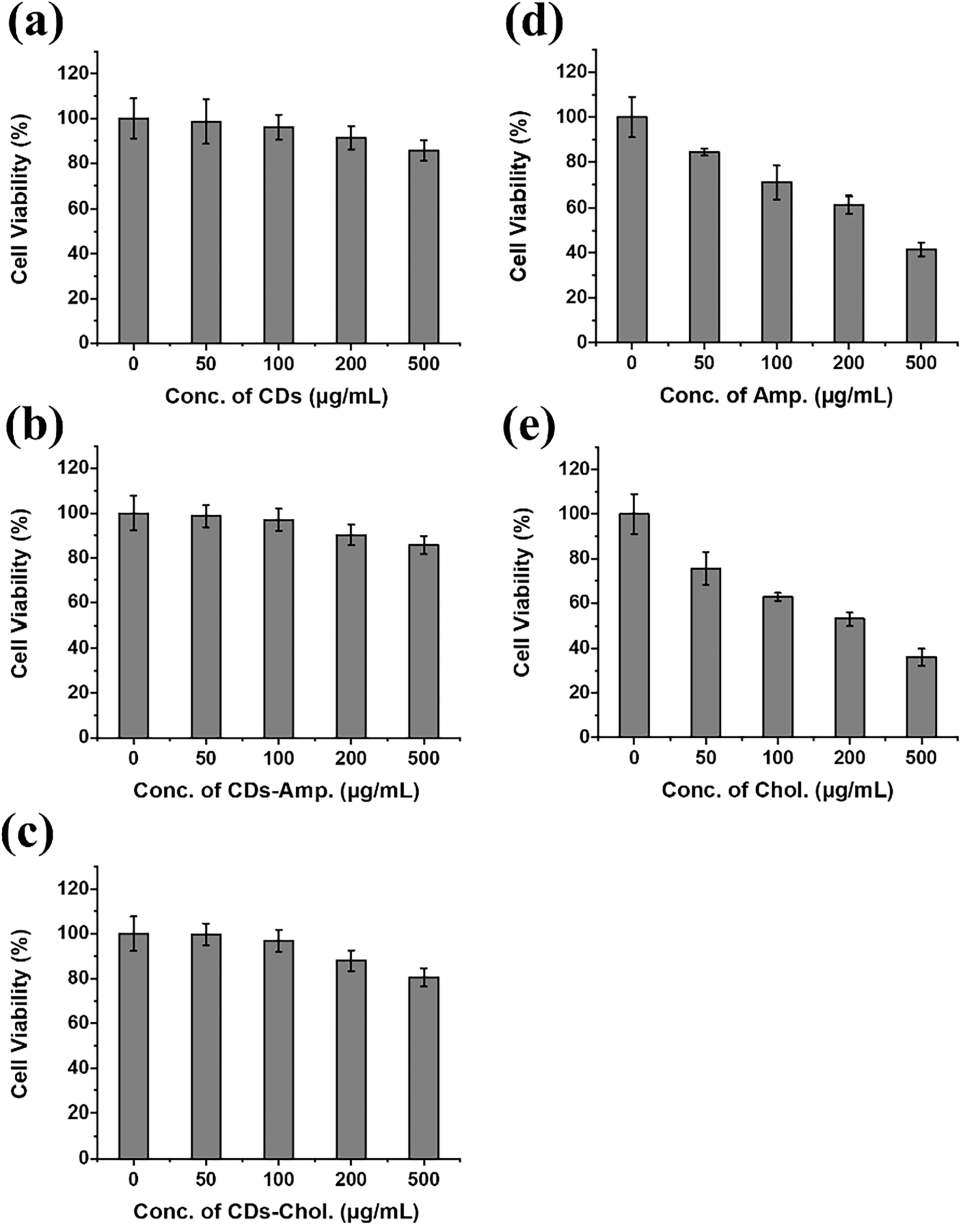
Cell viability at various concentrations of (a) CDs, (b) CDs-ampicillin, and (c) CDs-chloramphenicol nanocomposites, (d) pristine ampicillin, and (e) chloramphenicol alone. Statistical analysis was assessed using Student’s *t* test (*p* > 0.05). Data are presented as mean ± standard error of the mean (SEM), *n* = 3.

The cytotoxicity assessment of CDs and their antibiotic-conjugated derivatives was carried out using the MTT assay on HeLa cells, revealing encouraging biocompatibility outcomes. Pristine CDs demonstrated minimal adverse effects on cell viability, maintaining nearly complete cellular health even at concentrations as high as 500 μg/mL, underscoring their safety for biological applications. Upon modification with antibiotics, specifically ampicillin and chloramphenicol, the nanocomposites exhibited a slight reduction in cell viability. This decline is consistent with the expected biological activity of the antibiotic agents, yet the overall cytotoxic impact remained significantly lower than that of the free drug forms. These results suggest that while antibiotic functionalization introduces a modest increase in cytotoxicity, the nanocomposites still offer a safer alternative for therapeutic use, combining antimicrobial efficacy with reduced cellular damage.

### 3.3 Antibiotic Activity Against Multidrug-Resistant Bacteria

The loading content was calculated as the weight percentage of the antibiotic agent in the total nanocomposite mass, while drug loading efficiency (DLE) represented the proportion of initially added antibiotic that was conjugated to the CDs. The observed DLC values ranged between 15 and 20%, confirming effective antibiotics incorporation. The antibacterial efficacy of the CDs-chloramphenicol and CDs-ampicillin nanoparticles was systematically evaluated against two model bacterial strains: *E. coli* (ATCC 25922), a Gram-negative bacterium, and *Staphylococcus aureus* (ATCC 25923), a Gram-positive bacterium. The experimental procedure involved treating the bacterial cultures with aqueous dispersions of the nanoconjugates in double-distilled water (DDW) with the help of mild sonication to ensure uniform dispersion and optimal interaction between the nanoparticles and the bacterial cells. The antibacterial performance was assessed after 50 min of exposure. For *S. aureus*, the CDs-chloramphenicol and CDs-ampicillin nanoparticle complexes caused a significant reduction in the bacterial viability, demonstrating a 3.78-log reduction in colony-forming units (CFUs), far surpassing the antibacterial effects observed with other control treatments (no more than 1.5-log reduction). Furthermore, these complexes seem to be highly promising nanomaterials for combating the multidrug-resistant bacteria hospital strains Dh5a–ampicillin R and BL21 chloramphenicol R. We observed a 4-fold killing rate of BL21 chloramphenicol R bacteria by CDs-chloramphenicol with respect to the same concentration of chloramphenicol drug and a 2-fold killing rate of Dh5a ampicillin R strain bacteria by CDs-Amp drugs with respect to ampicillin (Table 1). These results highlight the superior bactericidal effect of the nanoconjugates against hospital multidrug-resistant bacteria. The results of complete bacterial eradication, which are presented in Table 1, were obtained within just 25 min of exposure to the CDs-antibiotic nanoparticle complex. In comparison, treatments with individual CDs-chloramphenicol and CDs-ampicillin nanoparticle solutions in water resulted in a 3.05-log and 2.87-log reduction, respectively, indicating that the conjugation of either antibiotics with CDs in a single hybrid nanoparticle formulation has a synergistic or additive effect on antimicrobial efficacy. Notably, other control treatments, including free antibiotics or bare CDs, had minimal effect on bacterial survival under the same conditions.

**Table 1:** Results of the MIC (Minimal Inhibitory Concentration) Test (μg/mL) for Various Strains of Bacteria and Types of Treatment^a^

	bacteria		
treatment	Dh5a–ampicillin R	*E. coli* ATCC	BL21 chloramphenicol R
CDs-ampicillin	6000	11	
pristine ampicillin	>12,000	23	
pristine CDs	12,000		300
pristine chloramphenicol		9.3	150
CDs-chloramphenicol		9.3	37.5

a Results are expressed as mean ± SEM, *n* = 3.

Since reactive oxygen species (ROS) generation contributes to the antimicrobial activity of various nanomaterials, we employed electron spin resonance (ESR) spectroscopy to confirm its production in our case. Specifically, we analyzed the photoexcited samples using the DMPO/OH spin adduct, which revealed four distinct peaks with a hyperfine splitting constant of 14.9 G (aN=aH), as shown in Figure 8. These results indicate that ROS is generated in all CD-based samples and their antibiotic nanocomplexes, but not in the pristine forms of ampicillin and chloramphenicol. Notably, the signal intensity for ROS was significantly higher in CDs, CDs-chloramphenicol, and CDs-ampicillin compared to the pristine antibiotics. This comparison was made using equal molar amounts of CDs and antibiotics loaded onto the CDs. Therefore, we can attribute the enhanced ROS signals in the presence of nanocomposites to the increased number of defect sites on the CDs, which likely serve as active centers for antimicrobial action. Importantly, we ruled out the involvement of reactive nitrogen species, as the ESR baseline for CDs showed no distinct features. Based on these findings, we propose that the elevated ROS levels produced by the CDs-chloramphenicol and CDs-ampicillin nanocomposites overwhelm the cellular antioxidant defenses, leading to rapid cell damage and death, as summarized in Table 1.

**Figure 8:**
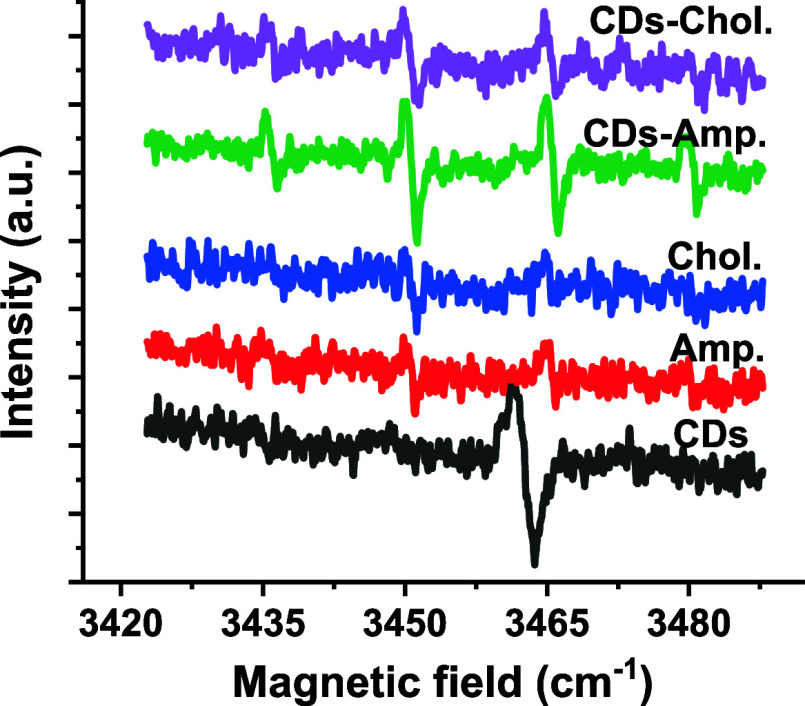
ESR spectra for ROS generated by CDs, CDs-chloramphenicol, and CDs-ampicillin compared to the pristine antibiotics chloramphenicol and ampicillin.

The synergistic effect of nanocomposites not only improves the therapeutic index but also helps to reduce the required antibiotic dose, potentially minimizing side effects and slowing the development of further resistance. As such, CDs-antibiotics represent a versatile and powerful platform for the development of next-generation antibacterial therapies aimed at overcoming the growing threat of antibiotic resistance. Overall, these findings demonstrate that the CDs-chloramphenicol and CDs-ampicillin nanoparticle complexes exhibit highly efficient and broad-spectrum antibacterial activity, with enhanced efficacy against both Gram-positive and Gram-negative multidrug-resistant bacteria. This suggests their strong potential as an alternative therapeutic approach to combating antibiotic-resistant infections. The enhanced effect of 2–4-fold refers to a reduction in the minimal inhibitory concentration (MIC) values when antibiotics are conjugated to CDs. For example, CDs-ampicillin reduced the MIC against *E. coli* ATCC from 23 to 11 μg/mL and CDs-chloramphenicol reduced the MIC against *E. coli* BL21 from 150 to 37.5 μg/mL.

Development of new antibacterial agents and diagnosis of infections caused by multidrug-resistant bacterial strains (such as *E. coli* ATCC and Dh5α–ampicillin resistant) requires accurate identification of bacterial strains. In this study, we evaluated the fabricated CDs-chloramphenicol and CDs-ampicillin nanoparticle complexes for their potential in bioimaging of multidrug-resistant bacteria (Figure 9). The results demonstrate that these nanoparticle complexes can serve as effective probes, enabling visualization of their entry into resistant bacterial cells. They exhibit strong fluorescence signals and further allow differentiation between live and dead bacterial cells.

**Figure 9:**
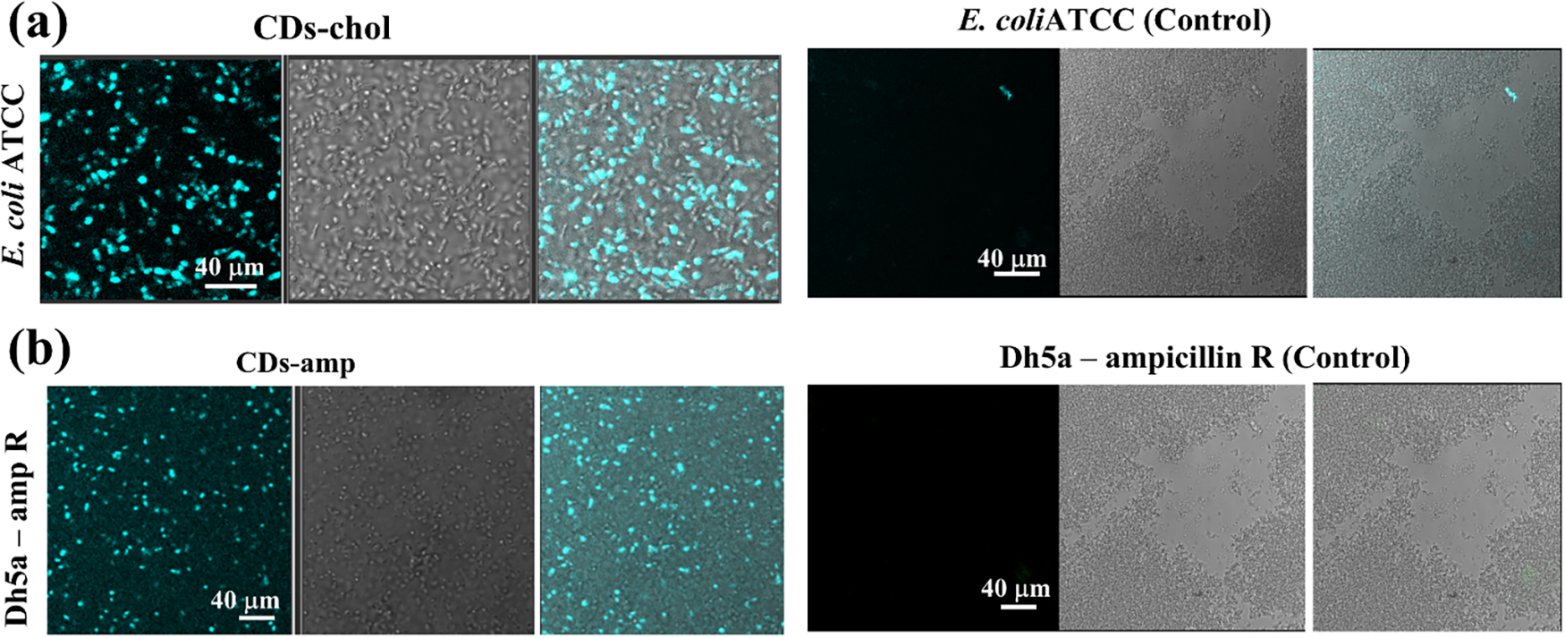
Live cell imaging of multidrug-resistant bacterial cells and types of treatment: (a) *E. coli* ATCC with or without (control) CDs-Chol and (b) Dh5a ampicillin R with or without (control) CDs-Chol treatment for ∼6 h.

Overall, the combination of potent antibacterial efficacy (2–4 times more effective against multidrug-resistant bacteria) and low cytotoxicity strongly suggests that the CDs-ampicillin and CDs-chloramphenicol nanocomposites are promising and biocompatible platforms for the development of next-generation antibiotic delivery systems. These carbon-dot-based nanohybrids not only enhance the antibacterial potential of conventional antibiotics but also offer a flexible and safe alternative for various practical applications. For instance, in topical or external treatments, such as skin infections, the nanocomposites could be applied directly to the affected area. When combined with UV illumination, these nanomaterials can act as photocatalysts to generate reactive oxygen species (ROS), particularly hydrogen peroxide (H_2_O_2_), which is known to effectively penetrate bacterial cell walls and induce oxidative damage to intracellular components, resulting in rapid bacterial cell death. Moreover, such nanocomposites may be utilized in environmental applications, such as water purification and surface disinfection, where their photocatalytic activity under light exposure can further enhance the antimicrobial action. It is important to note, however, that while H_2_O_2_ can diffuse across bacterial membranes, other ROS such as superoxide anions (O_2_^–^) and hydroxyl radicals (^•^OH) are negatively charged and highly reactive, making it unlikely for them to cross the similarly negatively charged bacterial cell membrane. Consequently, their antibacterial effect is predominantly localized at the membrane interface. This highlights that the primary mode of action for these nanocomposites likely involves membrane-level interactions such as disruption of membrane integrity, oxidative stress at the surface, and increased permeability rather than intracellular damage. Therefore, further studies focusing on the surface-level mechanisms of bacterial inactivation could provide deeper insights into optimizing these CD-based antibiotic systems for clinical and environmental applications.

## 4 Conclusions

Although conjugation of CDs and antibiotic molecules has been reported in the literature, the novelty of this work includes two aspects: (a) Formation of the nanocomposites by two separate sonochemical steps; (b) conjugation of two other antibiotic agents, chloramphenicol and Ampicillin, to CDs, forming CDs-chloramphenicol and CDs-ampicillin, which were found to be more efficient against multidrug-resistant bacteria. Several analytical methods were used to confirm the conjugation of the antibiotic molecules onto the fluorescent carbon dot surfaces: UV–vis spectroscopy showed a great similarity between the spectra of pristine Amp. to that of CDs-Amp., whereas the spectrum for pristine CDs was different. The same was observed for Chlor. and CDs-Chlor. Fluorescence spectroscopy revealed different spectra for the two kinds of nanocomposites compared with their pristine components. FTIR spectra of CDs-Amp. and CDs-Chlor. look as a superposition of the spectra of their pristine components. Moreover, the improvement in the antibiotic activity of the product implies variations in its composition and structure with respect to the pristine antibiotic molecules. The resulting nanocomposites exhibited excellent aqueous dispersibility, high colloidal stability, and uniform nanoscale dimensions. The improved bioactivity was accomplished by creating more active sites on the CDs, serving as ROS generation sites, thus enhancing the antibiotic agents. Most notably, the CDs-antibiotic nanohybrids demonstrated potent antibacterial efficacy against both Gram-positive (*S. aureus*) and Gram-negative (*E. coli*) strains, including those known to exhibit multidrug resistance. The enhanced antibacterial effect can also be attributed to synergistic interactions between the CDs and the conjugated antibiotics, facilitating membrane-level disruption and possibly reactive oxygen species (ROS)-mediated bacterial killing. Taken together, these findings suggest that the engineered CDs, chloramphenicol, and CDs, ampicillin nanoparticles offer a promising and biocompatible nanoplatform for antibacterial applications. Their potential utility spans across biomedical fields such as tropical infection treatment, wound healing, and as antimicrobial agents in water purification systems. Future work may focus on in vivo studies, mechanistic investigations, and integration into practical delivery systems to further develop these nanocomposites into viable antimicrobial therapies.
